# Trends in Pesticide Concentrations in Streams of the Western United States, 1993-2005^1^

**DOI:** 10.1111/j.1752-1688.2010.00507.x

**Published:** 2011-04

**Authors:** Henry M Johnson, Joseph L Domagalski, Dina K Saleh

**Keywords:** pesticides, monitoring, time series analysis, watershed management, TMDL, salmon

## Abstract

Trends in pesticide concentrations for 15 streams in California, Oregon, Washington, and Idaho were determined for the organophosphate insecticides chlorpyrifos and diazinon and the herbicides atrazine, s-ethyl diproplythiocarbamate (EPTC), metolachlor, simazine, and trifluralin. A parametric regression model was used to account for flow, seasonality, and antecedent hydrologic conditions and thereby estimate trends in pesticide concentrations in streams arising from changes in use amount and application method in their associated catchments. Decreasing trends most often were observed for diazinon, and reflect the shift to alternative pesticides by farmers, commercial applicators, and homeowners because of use restrictions and product cancelation. Consistent trends were observed for several herbicides, including upward trends in simazine at urban-influenced sites from 2000 to 2005, and downward trends in atrazine and EPTC at agricultural sites from the mid-1990s to 2005. The model provided additional information about pesticide occurrence and transport in the modeled streams. Two examples are presented and briefly discussed: (1) timing of peak concentrations for individual compounds varied greatly across this geographic gradient because of different application periods and the effects of local rain patterns, irrigation, and soil drainage and (2) reconstructions of continuous diazinon concentrations at sites in California are used to evaluate compliance with total maximum daily load targets.

## Introduction

Across the United States (U.S.), federal, tribal, state, and local agencies are acting to reduce pesticide transport to the aquatic environment because of the adverse effects to stream ecology and human health posed by pesticides. In California and the Pacific Northwest (Oregon, Washington, and Idaho), economically, ecologically, and culturally important fish populations are in serious decline. Toxic compounds, including pesticides, are routinely included among the factors contributing to the declines (Bennett, 2005; Fresh *et al.*, 2005). Of particular concern are populations of delta smelt (*Hypomesus transpacificus*) and Chinook salmon (*Oncorhynchus tshawytscha*) in the Sacramento River and San Joaquin River systems and multiple species of Pacific salmon (*Oncorhynchus* spp.) in the Columbia River/Snake River system (Myers *et al.*, 1998). A number of investigations have demonstrated detrimental effects of environmentally relevant concentrations of pesticides to salmonids (Scholz *et al.*, 2000; Tierney *et al.*, 2008; Laetz *et al.*, 2009). Although no similar toxicity studies exist for delta smelt, Kuivila and Moon (2004) documented the occurrence of pesticide mixtures in the delta smelt's habitat during their highly susceptible early life stages and Domagalski and Munday (2003) documented pesticides being delivered by the San Joaquin River to delta smelt habitat during critical life stages. The relative contribution of pesticide toxicity to decreasing fish populations compared with other stressors such as flow regulation, habitat loss, and climate change in these systems is a subject of active debate and research (Bennett, 2005; Fresh *et al.*, 2005; Loge *et al.*, 2005).

Organophosphate (OP) insecticides are the most actively managed group of pesticides in California and the Pacific Northwest. In part, this is because of their toxicity to aquatic invertebrates (Kuivila and Foe, 1995; de Vlaming *et al.*, 2000) which are important prey organisms for fish, and also to their effect on salmonid behavior (Scholz *et al.*, 2000; Sandahl *et al.*, 2005). In 2004, all urban uses of the OP insecticide diazinon were eliminated and significant restrictions were placed on its agricultural use, including cancelation of its use on many crops, a reduction in application rates, a reduction in the number of allowable annual applications, elimination of aerial applications, and restrictions on the methods of application (USEPA, 2006a). At the state and regional level, efforts to prevent OP insecticides from reaching streams include: total maximum daily load (TMDL) plans by the CalEPA (2003, 2005, 2006a,b), listing of stream reaches in Washington as impaired under Section 303(d) of the Clean Water Act (Washington State Department of Ecology, 2009), formation of a multiagency pesticide management effort in Oregon (Oregon Department of Agriculture, Oregon Department of Environmental Quality, Oregon Department of Forestry and Oregon Department of Human Services, 2008), and a ruling by the U.S. District Court for the Western District of Washington in the case of Washington Toxics Coalition *v.* EPA (USEPA, 2004a), which restricted the application of 26 pesticides (including 12 OP insecticides) adjacent to streams used by salmon that are listed as threatened or endangered under the Endangered Species Act (USFW, 2009).

Other pesticides have not been subject to the same intense regulatory pressure as the OP insecticides, however mounting evidence from field and laboratory studies indicate that some of these compounds have sublethal or long-term health effects of concern. Triazine herbicides such as atrazine, simazine, and hexazinone, are applied in large quantities and have a variety of uses across the landscape in California and the Pacific Northwest (USDA, 2009a). Triazines have been shown to be synergists, enhancing the lethality of some insecticides (Lydy and Austin, 2004; Banks *et al.*, 2005; Trimble and Lydy, 2006) and estrogenic activity of atrazine and simazine have been linked to hormonal and developmental changes in laboratory test animals (USEPA, 2006b). Other widely used herbicides in the study area include metolachlor which is classified as a potential carcinogen by the U.S. Environmental Protection Agency (USEPA) (1995) and s-ethyl diproplythiocarbamate (EPTC) which has been linked to degeneration of the heart and nervous system (USEPA, 1999).

A key indicator of the success of efforts to reduce the movement of pesticides into streams would be a decrease in measured instream pesticide concentrations. However, short- and long-term hydrologic variability can obscure trends in concentration, making it difficult to ascertain the success or failure of mitigation efforts by monitoring alone. In surface water, parametric multivariate regression models have been used successfully to estimate trends for cations, anions, nutrients, suspended sediment, and dissolved organic carbon (Saleh *et al.*, 2003; Langland *et al.*, 2004; Vecchia, 2005; Sprague *et al.*, 2007). These models include terms that account for hydrologic variability (stream discharge and seasonality) and monotonic changes in concentration or load over time. The use of these models for pesticides has met with limited success, however (Frey, 2001). Two issues confound the use of these models for pesticides, including (1) the representation of the seasonal occurrence of pesticides in the stream as a simple two-term Fourier series composed of a single sine and a cosine function (Cohn *et al.*, 1992a; Goolsby *et al.*, 2001; Schilling, 2002) and (2) an often weak relation between pesticide concentration and stream discharge. Recently, Vecchia *et al.* (2008) developed a parametric regression model that addresses these issues. The particularly novel component of the model developed by Vecchia *et al.* (2008) is the representation of pesticide seasonality which was formulated as a pulse with decay rather than a sinusoidal oscillation generated by the simple Fourier series. Terms in the seasonality function allow the user to control the location, duration, and decay of the annual peak or peaks in pesticide concentration, which enables the modeler to approximate the observed pesticide seasonality in nearly any stream.

This paper describes trends in pesticide concentrations in 15 streams in California, Oregon, Washington, and Idaho using data collected by the U.S. Geological Survey (USGS) National Water-Quality Assessment (NAWQA) Program between 1992 and 2005. Seven pesticides were selected for trend analysis based on overlap in use and occurrence at the 15 sites. Monotonic trends in pesticide concentrations (corrected to remove hydrologic variability) were calculated using a parametric multiple linear regression model that includes the seasonal pulse term of Vecchia *et al.* (2008). Where possible, trends are discussed in relation to known regulatory and land use changes.

## Materials and Methods

### Study Sites

**FIGURE 1:**
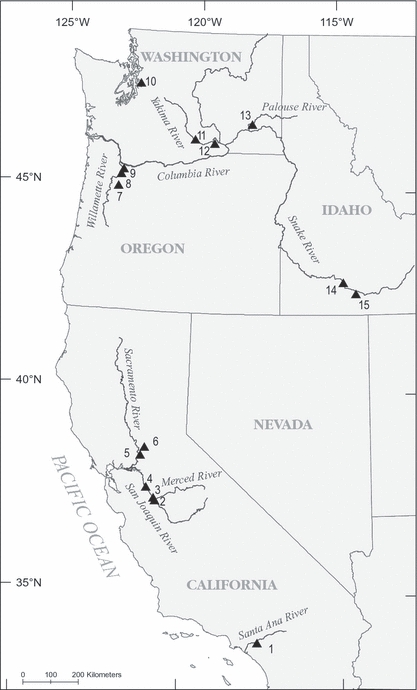
Map Showing Location of Sampled Streams. Triangles represent the approximate location of the sampling site. Small streams cannot adequately be displayed at the regional scale and are not shown.

Sampling sites were located in a variety of land use settings including small catchments that were primarily agricultural or urban, and larger catchments with a mixture of land use or cover (Figure 1). Catchment areas varied from 27 km^2^ to 92,942 km^2^. Catchment size, mean daily discharge, land use, and annual precipitation for each site are shown in Table 1. In general, all sites are characterized by hot, dry summer months and relatively wet winters, although the total annual precipitation varied greatly among sites. At many sites, snowmelt originating in higher elevation portions of the catchment contributes to stream flow at the sampling site during part of the year; at the sampling sites, themselves, however, most precipitation falls as rain.

**TABLE 1:** Characteristics of Modeled Sites

Map Reference Number	Site Name	Short Name	USGS Site Number	Land Use				Mean Daily Stream Discharge (m^3^/s)	Catchment Drainage Area (km^2^)	Mean Annual Precipitation, 1971-2000 (mm)
							
				A	U	F	O			
1	Santa Ana River below Prado Dam, California	Santa Ana R, CA	11074000	8.6	22.8	20.5	48.1	11.2	3,726.5	273
2	Merced River at River Road Bridge near Newman, California	Merced R, CA	11273500	13.5	1.0	48.1	37.4	22.4	3,611.0	322
3	Orestimba Creek at River Road near Crows Landing, California	Orestimba Cr, CA	11274538	95.3	0.3	0.0	4.5	1.3	27.9	296
4	San Joaquin River near Vernalis, California	San Joaquin R, CA	11303500	22.5	2.4	33.9	41.2	129.8	19,029.9	322
5	Sacramento River at Freeport, California	Sacramento R, CA	11447650	13.1	1.9	52.4	32.7	737.9	61,692.7	431
6	Arcade Creek near Del Paso Heights, California	Arcade Cr, CA	11447360	0.1	92.5	3.3	4.1	0.6	81.5	501
7	Zollner Creek near Mount Angel, Oregon	Zollner Cr, OR	14201300	95.2	1.1	2.6	1.1	0.6	38.9	1,218
8	Fanno Creek at Durham, Oregon	Fanno Cr, OR	14206950	6.2	57.5	28.4	7.8	1.3	80.7	944
9	Willamette River at Portland, Oregon	Willamette R, OR	14211720	23.5	3.9	65.3	7.3	978.5	28,936.9	1,018
10	Thornton Creek near Seattle, Washington	Thornton Cr, WA	12128000	0.0	88.3	7.7	4.0	0.3	29.2	944
11	Granger Drain at Granger, Washington	Granger Dr, WA	12505450	59.2	3.3	0.2	37.3	1.0	159.9	205
12	Yakima River at Kiona, Washington	Yakima R, WA	12510500	15.0	2.0	36.3	46.7	82.2	14,536.2	205
13	Palouse River near Hooper, Washington	Palouse R, WA	13351000	72.6	1.4	9.6	16.4	18.0	6,378.8	438
14	Snake River at King Hill, Idaho	Snake R, ID	13154500	17.4	0.4	15.9	66.3	279.7	92,942.4	282
15	Rock Creek above Highway 30/93 Crossing at Twin Falls, Idaho	Rock Cr, ID	13092747	21.8	1.5	3.3	73.5	3.2	664.4	282

Notes: A, agriculture; U, urban; F, forest; O, other; USGS, U.S. Geological Survey. Values of land use are reported as percent of catchment drainage area.

For inclusion in this study, sites were required to have at least three years of pesticide sampling, each with a minimum of six samples per year. Daily stream flow for the entire modeling period also was required and was obtained from the USGS National Water Information System (NWIS).

### Source of Pesticide Data

Pesticide concentration data for the 15 study sites were obtained from Martin (2009). Martin's dataset was compiled from samples collected by the USGS NAWQA Program and National Stream Quality Accounting Network (NASQAN) Program between 1992 and 2006. Data collection techniques for the two programs are similar. Briefly, stream water was collected using depth and width integrating sampling techniques (USGS, 2006). Sample collection and storage vessels were composed of polytetrafluoroethylene or glass. On the same day of sampling, water was filtered through 0.7 micron precombusted glass fiber filters held in an aluminum filter housing. Filtrate was collected in precombusted glass bottles and shipped on ice overnight to the USGS National Water Quality Laboratory (NWQL) in Denver, Colorado, for analysis. Pesticide analyses were conducted by gas chromatography/mass spectroscopy with selected ion monitoring using the methods of Zaugg *et al.* (1995). For the 15-year data collection period, a consistent minimum reporting level was determined for each pesticide compound (Martin, 2009), a bias correction was applied to the concentration values to account for changes in laboratory recovery (Martin *et al.*, 2009), and a consistent method of rounding was applied to the concentration values (Martin, 2009). Recognizing the potential for correlation among samples closely spaced in time and the implications for the development of trend models, Martin (2009) added an attribute to the database that allows users to create a subset of the data that has no more than one sample per calendar week (Sunday to Saturday). This feature was used to produce the datasets for this study.

For a pesticide to be considered for trend analysis two criteria were required to be met at a minimum of four sites: (1) a pesticide was required to have at least 20 uncensored concentration values and (2) at least 20% of all the concentration values had to be uncensored. Censoring is common in pesticide data and refers to a pesticide concentration reported as less than some value; in this paper the censoring value is the *maximum long-term method detection level* of Martin (2009). Censoring occurs because the analyzing laboratory is unable to detect the pesticide or quantify its concentration in the sample. The censoring level is set by the laboratory and is based upon analyses of samples into which known quantities of pesticides have been added. A thorough discussion of censoring related to this dataset is provided by Martin (2009). Of the 52 pesticides available from Martin (2009), atrazine, chlorpyrifos, diazinon, EPTC, metolachlor, simazine, and trifluralin met our criteria for trend analysis. Pesticide degradation products were not considered for this analysis because of difficulties in comparing those compounds with changing uses of the parent product.

A common period of record is required for trends to be comparable among sites. For the purposes of this paper, a comparison between two sites was considered valid if (1) the first samples at each site were collected within 365 days of one another and (2) the last samples at each site were collected within 365 days of one another. The first samples collected at the 15 study sites varied between 1992 and 2000 because of the staggered sampling design of the USGS NAWQA program. The pesticide concentration data were analyzed to identify the period of record that would enable the greatest number of sites to be compared. Two time periods were chosen for trend analysis. The first trend period began in 2000 or 2001 and ended in 2004 or 2005 and provided a common period for comparison of trends among all sites. The mean date of the first sample used for this trend period was June 2000 and the mean date of the last sample was May 2005. To simplify the ensuing discussion, this period is deemed to have begun in 2000 and ended in 2005. For the second trend comparison, sites were grouped by catchment size and land use and the earliest start date for each group was determined. Some sites were eliminated from the latter comparison because the 365-day criterion was not met. The second trend period began either in 1992, 1993, 1994, or 1996 and ended in 2004 or 2005. The mean date of the first sample used for this trend period was August 1996 at small urban sites, February 1993 at small agricultural sites, and August 1993 at large mixed land use sites. The mean date of the last sample for the three groups was between April and September 2005. As with the shorter trend period, the longer trend periods will be referenced in the discussions using their mean dates. The time-series pesticide concentration data from Martin (2009) were trimmed to match the trend periods and selection criteria discussed earlier in this paragraph. Only concentration data from the beginning or ending of a time series were deleted – no data were removed from the middle of a time series at a site. The first sample, last sample, number of concentration values, and number of censored values for each site and each trend period are shown in Table 2. The maximum long-term method detection level for each pesticide also is provided.

**TABLE 2:** Period of Record and Number of Measured Pesticide Samples for Each Site. Format for pesticide observations is: total number of observations (number of uncensored observations)

					Number of Pesticide Observations						
					
					Atrazine	Chlorpyrifos	Diazinon	EPTC	Metolachlor	Simazine	Trifluralin
											
Short Name	Trend Period	Date of First Sample	Date of Last Sample	Period of Record (years)	Maximum LT-MDL = 0.004 μg/l	Maximum LT-MDL = 0.003 μg/l	Maximum LT-MDL = 0.003 μg/l	Maximum LT-MDL = 0.002 μg/l	Maximum LT-MDL = 0.006 μg/l	Maximum LT-MDL = 0.006 μg/l	Maximum LT-MDL = 0.005 μg/l
**Small streams, urban**											
Santa Ana R, CA	1	7/13/2000	6/13/2005	4.92	43 (17)	-	43 (6)	-	-	43 (0)	-
Arcade Cr, CA	1	1/17/2001	7/27/2005	4.52	-	55 (28)	55 (0)	-	54 (24)	55 (16)	-
Fanno Cr, OR	1	2/20/2001	8/2/2005	4.45	70 (7)	71 (56)	71 (19)	-	71 (50)	71 (9)	-
Thornton Cr, WA	1	1/11/2001	7/25/2005	4.53	-	-	-	-	-	54 (39)	-
Arcade Cr, CA	2	11/26/1996	9/28/2005	8.84	-	88 (38)	88 (0)	-	87 (38)	88 (26)	-
Thornton Cr, WA	2	4/11/1996	9/20/2005	9.44	-	-	-	-	-	99 (73)	-
**Small streams, agricultural**											
Orestimba Cr, CA	1	2/25/2000	6/22/2005	5.32	87 (35)	92 (37)	92 (22)	92 (28)	92 (12)	92 (4)	92 (14)
Zollner Cr, OR	1	4/10/2000	6/9/2005	5.16	80 (0)	80 (11)	80 (41)	80 (12)	80 (0)	80 (0)	80 (59)
Granger Drain, WA	1	6/21/2000	9/21/2004	4.25	96 (1)	-	-	-	-	96 (69)	96 (57)
Rock Cr, ID	1	3/8/2000	7/20/2005	5.37	80 (22)	-	-	63 (33)	-	-	-
Orestimba Cr, CA	2	8/5/1992	9/7/2005	13.09	169 (90)	174 (68)	173 (48)	174 (61)	174 (32)	174 (8)	174 (36)
Zollner Cr, OR	2	8/3/1993	8/3/2005	12.00	138 (0)	138 (36)	138 (58)	138 (28)	138 (0)	138 (0)	138 (99)
Rock Cr, ID	2	4/20/1993	9/6/2005	12.38	167 (30)	-	-	150 (75)	-	-	-
**Large streams, mixed land use**											
Merced R, CA	1	2/25/2000	6/22/2005	5.32	-	92 (59)	92 (67)	90 (69)	-	92 (41)	-
San Joaquin R, CA	1	2/23/2000	6/22/2005	5.33	89 (39)	93 (43)	93 (22)	92 (19)	93 (24)	93 (8)	93 (44)
Sacramento R, CA	1	2/24/2000	7/21/2005	5.40	-	-	59 (32)	-	59 (36)	59 (29)	-
Willamette R, OR	1	3/14/2000	6/7/2005	5.23	74 (15)	-	-	71 (54)	74 (48)	74 (28)	-
Yakima R, WA	1	1/11/2001	6/15/2005	4.42	58 (14)	58 (45)	-	-	-	58 (43)	-
Palouse R, WA	1	3/7/2000	8/2/2004	4.41	61 (35)	-	-	-	-	61 (38)	-
Snake R, ID	1	3/21/2000	7/19/2005	5.33	62 (20)	-	-	50 (33)	-	-	-
Merced R, CA	2	6/1/1993	6/22/2005	12.06	-	159 (98)	159 (110)	157 (115)	-	159 (61)	-
San Joaquin R, CA	2	6/1/1993	6/22/2005	12.06	163 (90)	167 (83)	167 (44)	166 (35)	167 (55)	167 (14)	167 (89)
Willamette R, OR	2	9/1/1993	6/7/2005	11.76	155 (20)	155 (124)	155 (115)	152 (121)	155 (72)	155 (41)	-
Palouse R, WA	2	6/3/1993	8/2/2004	11.16	129 (51)	-	-	-	-	129 (72)	-
Snake R, ID	2	5/24/1994	7/19/2005	11.15	106 (32)	-	-	94 (57)	-		-

Notes: R, River; Cr, Creek; maximum LT-MDL is that of Martin (2009); “-” indicates too many censored values for trend analysis. Dates are mm/dd/yy notation.

### Source of Precipitation Data

Precipitation data were obtained from the National Weather Service (NOAA, 2009). A weather station located in the catchment draining to the sampling site was used when possible. Some small catchments did not have weather stations or the station record was inadequate, and in these cases, a comparable, nearby site was used. In all cases, a weather station was selected that was representative of precipitation conditions in the portion of the catchment where pesticides are commonly used and where rainfall runoff from the surrounding land was likely to reach the sampling site in less than five days.

### Model Development

Trends in pesticide concentrations were estimated using the coefficient of the linear time term in a log-linear load model using the computer code LOADEST (Runkel *et al.*, 2004). LOADEST was implemented within the S-PLUS statistical software package (S-PLUS version 7.0; Insightful Corporation) using the USGS S-PLUS interface to LOADEST (USGS library for S-PLUS, version 7.0, for Windows – Release 3.1). LOADEST includes routines that correct for retransformation bias that results from the model being formulated as a logarithmic function. Model coefficients were estimated using the adjusted maximum likelihood estimator (AMLE) developed by Cohn (1988; Cohn *et al.*, 1992b). The AMLE procedure treats censored values probabilistically rather than relying on fixed-value substitution.

Following the notation of Runkel *et al.* (2004), the general form of the load model is: 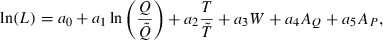
(1)where ln() is the natural logarithm function, *L* is the constituent load (kg), *Q* is the daily stream flow (cubic meters per second), *T* is decimal time (years), 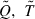 are centering variables as defined by Cohn *et al.* (1992a), *W* is a function that describes the annual fluctuation in the amount of a particular pesticide in a stream catchment available for transport (unitless), *A*_*Q*_ is antecedent flow (cubic meters per second), *A*_*P*_ is antecedent precipitation (unitless), and *a*_0_, *a*_1_,…, *a*_5_ are scalar coefficients.

The seasonality function (*W)* was developed by Vecchia *et al.* (2008). Mathematically, *W* is complex and is not amenable to a brief summary, so the reader is referred to Vecchia *et al.* (2008) for the derivation and related discussion. Conceptually, *W* empirically describes the annual cycle of pesticide occurrence in a stream. The best fit for *W* is developed by fitting two parameters, ω and ϕ, and the location within year to a time series of measured pesticide concentrations. The vector ω approximates the monthly inputs of a pesticide in the catchment. The scalar ϕ approximates the residence time of a pesticide in the stream catchment. ϕ condenses various important transport processes such as export, sorption, microbial degradation and mineralization, photodegradation, plant uptake, volatilization, and soil slope and permeability into a single holistic value useful in describing the retention and mobility of the pesticide in the stream and catchment being modeled. No single transformation process can be inferred from ϕ, however. The value of 12/ϕ approximates the half-life (in months) of the pesticide in the catchment.

**FIGURE 2:**
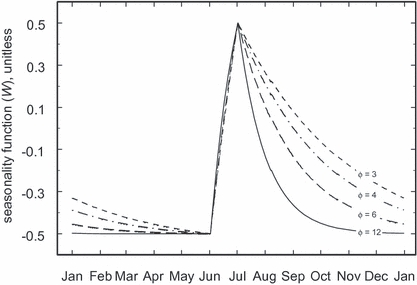
Seasonality Function (*W*) for Model 1 and All Values of ϕ (12, 6, 4, 3).

*W* can take on a variety of shapes depending upon the values of ω and ϕ used to define it. Fourteen basic seasonal shapes were defined for this modeling effort (Table 3). Each model, numbered 1-14, corresponds to a vector of ω (monthly inputs) and takes one of four values of ϕ. The resulting curves repeat on an annual cycle and are of unit size, varying from −0.5 to 0.5. As an example, the shape of *W* produced by Model 1 (one month of the year with pesticide input) with each of the four values of ϕ is shown in Figure 2.

**TABLE 3:** Model Forms of the Seasonality Function (*W*), and Values of ϕ and ω Used to Parameterize *W*

Model Number	ϕ	ω_1_	ω_2_	ω_3_	ω_4_	ω_5_	ω_6_	ω_7_	ω_8_	ω_9_	ω_10_	ω_11_	ω_12_
1	12, 6, 4, 3	0	0	0	0	0	1	0	0	0	0	0	0
2	12, 6, 4, 3	0	0	0	0	0	1	1	0	0	0	0	0
3	12, 6, 4, 3	0	0	0	0	0	1	1	1	0	0	0	0
4	12, 6, 4, 3	0	0	0	0	0	1	1	1	1	0	0	0
5	12, 6, 4, 3	0	0	0	0	1	1	1	1	1	0	0	0
6	12, 6, 4, 3	1	1	1	1	1	1	1	1	1	0	0	0
7	12, 6, 4, 3	0	0	1	1	1	0	0	0	1	0	0	0
8	12, 6, 4, 3	0	0	1	1	1	0	0	0	0	1	0	0
9	12, 6, 4, 3	0	0	1	1	1	0	0	0	0	0	1	0
10	12, 6, 4, 3	0	0	1	1	1	0	0	0	0	0	0	1
11	12, 6, 4, 3	0	0	1	1	1	0	0	0.75	0.75	0	0	0
12	12, 6, 4, 3	0	0	1	1	1	0	0	0	0.75	0.75	0	0
13	12, 6, 4, 3	0	0	1	1	1	0	0	0	0	0.75	0.75	0
14	12, 6, 4, 3	0	0	1	1	1	0	0	0	0	0	0.75	0.75

For each stream and pesticide all possible forms of *W* were generated and fit to the measured concentration data, and the best fitting forms of *W* were determined using the Akaike's (1981) information criterion (AIC). Typically, multiple forms of *W* were identified as equally good representations of the seasonality in measured concentration data. The final form of *W* was selected by visually comparing each of the automatically selected best forms with the measured concentration data. Some forms of *W* retained by the AIC were rejected because they did not reflect known application or occurrence patterns at a site. Of the remaining forms, the one with the largest AIC was selected. The model number, the value of ϕ, and the location of the maximum value of *W* within the year are provided in Table 4 for each pesticide at each site.

**TABLE 4:** Model Number (from Table 3), Decay Term (ϕ), and Peak Timing (in decimal years,July 1 = 0.5) for Each Pesticide Model. Format is: model number/ϕ/peak location

Short Name	Atrazine	Chlorpyrifos	Diazinon	EPTC	Metolachlor	Simazine	Trifluralin
**Small streams, urban**							
Santa Ana R, CA	4/12/0.70	-	1/12/0.11	-	-	4/6/0.12	-
Arcade Cr, CA	-	5/3/0.10	4/3/0.22	-	3/6/0.29	4/4/0.12	-
Fanno Cr, OR	9/12/0.39	2/12/0.40	8/12/0.40	-	13/12/0.87	14/6/0.35	-
Thornton Cr, WA	-	-	-	-	-	7/12/0.25	-
**Small streams, agricultural**							
Orestimba Cr, CA	1/12/0.49	5/3/0.49	13/12/0.60	1/3/0.36	3/6/0.49	4/3/0.18	4/6/0.52
Zollner Cr, OR	2/3/0.33	4/6/0.09	12/12/0.45	12/6/0.45	8/4/0.92	13/12/0.42	2/3/0.95
Granger Dr, WA	2/4/0.44	1/12/0.27	-	-	-	6/3/0.34	2/6/0.38
Rock Cr, ID	4/3/0.68	-	-	1/6/0.42	-	-	-
**Large streams, mixed land use**							
Merced R, CA	-	11/6/0.18	2/12/0.10	4/3/0.16	-	2/12/0.15	-
San Joaquin R, CA	3/12/0.52	11/6/0.25	2/4/0.07	4/3/0.49	4/6/0.55	3/3/0.13	5/3/0.42
Sacramento R, CA	-	-	2/6/0.11	-	1/12/0.39	3/6/0.15	-
Willamette R, OR	1/3/0.96	1/3/0.95	3/6/0.48	1/12/0.43	7/6/0.04	1/4/0.95	-
Yakima R, WA	3/3/0.60	9/4/0.25	-	-	-	14/6/0.30	-
Palouse R, WA	6/4/0.40	-	-	-	-	10/4/0.33	-
Snake R, ID	4/4/0.71	-	-	3/4/0.45	-	-	-

Notes: R, River; Cr, Creek; Dr, Drain.

The antecedent flow, *A*_*Q*_, was implemented as described by Vecchia *et al.* (2008). It was calculated as the difference between the natural logarithm of mean daily stream discharge on the day of sample collection and the mean of the natural logarithm of mean daily stream discharge from the preceding 30 days up to and including the day of sample collection (Equation 2).
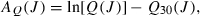
(2)where 
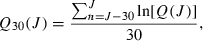
*Q*is the mean daily discharge, *J* is the Julian day of discharge measurement, and ln is the natural logarithm function.

The term had a range of −8.0 to +8.9 in the small urban and agricultural catchments and a range of −2.1 to +3.6 in the large, mixed land use catchments. Large positive values indicate that the discharge during sample collection was greater than the average conditions at the site during the last 30 days; conversely, a large negative value indicates that the discharge was much lower. *A*_*Q*_ provides a mechanism for the recent past to influence the prediction on the day of sample collection. The utility of such a mechanism is illustrated by the observations of Anderson *et al.* (1996) who found that concentrations of pesticides measured during a storm following a prolonged dry period were greater compared with concentrations measured in subsequent storms.

The antecedent precipitation, *A*_*P*_, is a ratio (unitless) that provides a measure of the intensity of recent precipitation compared with precipitation over the preceding 30 days. The term has a range of 0-1. It was calculated as:
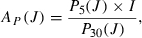
(3)where 
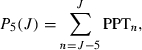

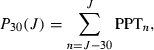
*I*=1 if *P*_5_(*J*) ≥20 mm, *I*=0 if *P*_5_(*J*) <20 mm, *J*is the Julian day of precipitation measurement, and PPT is total daily precipitation.

Analogous to *A*_*Q*_, *A*_*P*_ informs the model about the precipitation of the recent past.

### Computation of Trends

The pesticide load in a stream may change over time, for example, because of a regulatory change that permits its use on a particular crop grown in the catchment drained by that stream. Increasing or decreasing loads of a pesticide over the period of record of the input dataset are accounted for in the model by the decimal time term, *T* (Equation 1). The fitted coefficient of the decimal time term, *a*_2_, represents the slope of a line through time and can be interpreted as a trend through time. Trends were calculated using Equation (4).
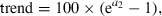
(4)where “trend” is expressed as a percent change in the pesticide load per year, e is the exponential, and *a*_2_ is the fitted coefficient of the decimal time term from Equation (1).

Trends were considered statistically significant when the *p*-value of the decimal time coefficient (*a*_2_) was 0.05 or less. The reported trends represent the average percent change per year during the period of time that was modeled.

A trend calculated using Equation (4) may be interpreted as either a trend in load or a trend in concentration. The dual interpretation stems from the fact that daily load on the left-hand side of Equation (1) can be recast as a function of concentration (Equation 5).
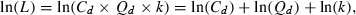
(5)where *C_d_* is the average daily concentration in micrograms per liter, *Q_d_* is the daily stream flow in cubic meters per second, and *k* is a units conversion constant.

A simple algebraic rearrangement of Equation (1) yields a concentration model in which the decimal time coefficient, *a*_2_, is identical to the load model. Because instream pesticide issues generally are concentration dependent (rather than load dependent), and because concentration data are more typically reported and discussed in the literature, discussions throughout the remainder of this paper will refer to concentrations rather than loads.

Trends calculated using Equation (4) do not represent trends in the measured instream concentrations. Rather, they provide an estimate of the trend one would observe in the absence of hydrologic variability (precipitation, runoff, irrigation, etc.) and seasonal differences in pesticide applications. By eliminating (in reality, minimizing) these confounding factors, the trend can be directly related to changes in the amount of pesticide used in a catchment or to changes in management practices that reduce the amount of pesticide reaching a stream.

### Model Evaluation

Each model was evaluated for goodness-of-fit by examining residuals for normality and heteroskedasticity; these diagnostics were acceptable for all significant models. The variance inflation factor (VIF) for each term in the model was examined to evaluate the potential for multicollinearity among model terms which could affect the sign and magnitude of the computed trends. VIFs were less than 10 in all 99 models having significant coefficients for the decimal time term, *T*, and less than 5 in 97 of the significant models. The two models with VIFs greater than 5 are discussed next.

The *Q* and *A*_*Q*_ terms in the model for diazinon at the Arcade Creek site, 2000-2005, had VIFs of 6.0 and 4.4, respectively. To evaluate the significance of the elevated VIF values, the six other possible models containing *Q* and *T* were developed: (1) *Q, T, A*_*Q*_*, A*_*P*_; (2) *Q, T, A*_*Q*_*, W*; (3) *Q, T, A*_*Q*_; (4) *Q, T, A*_*P*_*, W*; (5) *Q, T, A*_*P*_; (6) *Q, T, W*. The VIF and fitted coefficients from these models were compared with the original five-term model (Equation 1). This exercise showed that elevated VIF values are because of multicollinearity between *Q* and *A*_*Q*_ terms at this site. The multicollinearity did not affect the value or sign of the coefficient of the decimal time term, *T*, which varied between −0.54 and −0.56 in all models. The *p*-value of the *T* coefficient was less than 0.0001 in all models.

The *Q* and *W* terms in the model for atrazine at the Palouse River site, 2000-2005, had VIFs of 8.5 and 7.4, respectively. An analysis similar to the one done for Arcade Creek was done for this site, using the same model forms. The elevated VIFs are because of multicollinearity between the *Q* and *W* terms. As with the Arcade Creek site, the coefficient of the decimal time term, *T*, remained virtually unchanged in all model permutations, varying between 0.03 and 0.05. The *p*-value of the *T* coefficient was greater than 0.44 in all models.

## Results

Trends were calculated for two time periods. Short-term trend models were run for the period 2000-2005 (Table 5). This time period is the longest common period of data collection among all the sites and was done to facilitate a comparison among all sites. Long-term trend models were run at 10 of the 15 study sites: two small urban sites (1996-2005), three small agricultural sites (1993-2005), and five large mixed land use sites (1993-2005). Long-term trends are comparable only among similar site types, for example, small agricultural, because of differences in starting and ending dates of data collection at the various sites.

**TABLE 5:** Trends in Flow-Adjusted Concentrations of Pesticides at All Sites. Trends were calculated for the longest common periodrecord across all sites. Trends are calculated for models having a significant time coefficient (*p* ≤ 0.05). An entry of “NT” indicatesthat the *p*-value of the time coefficient was greater than 0.05. An entry of “-” indicates too many censored values for trend analysis

	Trend Period 1									
									
Site	Date of First Sample	Date of Last Sample	Period of Record (years)	Atrazine	Chlorpyrifos	Diazinon	EPTC	Metolachlor	Simazine	Trifluralin
**Small streams, urban**										
Santa Ana R, CA	7/13/2000	6/13/2005	4.92	NT	-	−25	-	-	59	-
Arcade Cr, CA	1/17/2001	7/27/2005	4.52	-	NT	−42	-	NT	22	-
Fanno Cr, OR	2/20/2001	8/2/2005	4.45	NT	NT	−29	-	NT	24	-
Thornton Cr, WA	1/11/2001	7/25/2005	4.53	-	-	-	-	-	NT	-
**Small streams, agricultural**										
Orestimba Cr, CA	2/25/2000	6/22/2005	5.32	NT	NT	−46	NT	20	NT	−18
Zollner Cr, OR	4/10/2000	6/9/2005	5.16	−12	NT	NT	−27	NT	NT	NT
Granger Drain, WA	6/21/2000	9/21/2004	4.25	−12	-	-	-	-	22	NT
Rock Cr, ID	3/8/2000	7/20/2005	5.37	NT	-	-	NT	-	-	-
**Large streams, mixed land use**										
Merced R, CA	2/25/2000	6/22/2005	5.32	-	NT	NT	NT	-	NT	-
San Joaquin R, CA	2/23/2000	6/22/2005	5.33	20	NT	−19	NT	NT	NT	NT
Sacramento R, CA	2/24/2000	7/21/2005	5.40	-	-	NT	-	NT	16	-
Willamette R, OR	3/14/2000	6/7/2005	5.23	NT	-	-	NT	NT	NT	-
Yakima R, WA	1/11/2001	6/15/2005	4.42	NT	27	-	-	-	NT	-
Palouse R, WA	3/7/2000	8/2/2004	4.41	NT	-	-	-	-	39	-
Snake R, ID	3/21/2000	7/19/2005	5.33	NT	-	-	NT	-	-	-

Notes: R, River; Cr, Creek. Dates are mm/dd/yy notation.

### Trends, All Sites, 2000-2005

Eighteen significant (*p*< 0.05) trends were identified for the period 2000-2005 (Table 5). Increasing pesticide concentrations were identified for nine cases and were equally balanced by decreasing pesticide concentrations for nine cases. The term “case” is used herein to refer to a unique pesticide-site pair, for example, atrazine at Orestimba Creek. Thirty-nine cases had no statistically significant trend.

Eleven of the 18 significant trends were associated with two pesticides – diazinon (5) and simazine (6). Downward trends in diazinon were identified at five sites and no significant trend occurred at three. No upward trends were identified for diazinon. The magnitude of the trends ranged from −19% to −46%/year. Downward diazinon trends were identified at all three small urban sites where sufficient data existed to build a model – Santa Ana River, California; Arcade Creek, California; and Fanno Creek, Oregon. One trend was at a small agricultural site (Orestimba Creek, California) and one at a large, mixed land use site (San Joaquin River, California).

Upward trends in simazine were identified at six sites and no significant trends were identified at seven. No downward trends in simazine were identified at any site. The magnitude of the trends ranged from +16% to +59%/year. As was seen with diazinon, upward trends were most consistently detected at the small urban sites, with three of the four sites having significant upward trends – Santa Ana River, California; Arcade Creek, California; and Fanno Creek, Oregon. One upward trend was identified at a small agricultural site (Granger Drain, Washington) and two upward trends were identified at large mixed land use sites (Sacramento River, California, and Palouse River, Washington).

Atrazine had three significant trends; 11 sites had models. Two of the trends were downward and occurred at small agricultural sites. One was upward and occurred at a large mixed land use site. The other four pesticides each had only one significant trend.

### Trends, Urban Sites, 1996-2005

Long-term trends were calculated at two urban sites: Arcade Creek, California, and Thornton Creek, Washington (Table 6). Four pesticides had sufficient data to construct models: chlorpyrifos, diazinon, metolachlor, and simazine; no significant trends were identified for either metolachlor or simazine.

**TABLE 6:** Trends in Flow-Adjusted Concentrations of Pesticides at Selected Sites. Trends were calculatedfor the longest common period of record across sites with similar upstream land use. Trends are calculated for models having a significant time coefficient (*p* ≤ 0.05). An entry of “NT” indicates that the *p*-value of the timecoefficient was greater than 0.05. An entry of “-” indicates too many censored values for trend analysis

	Trend Period 2									
									
Site	Date of First Sample	Date of Last Sample	Period of Record (years)	Atrazine	Chlorpyrifos	Diazinon	EPTC	Metolachlor	Simazine	Trifluralin
**Small streams, urban**										
Arcade Cr, CA	11/26/1996	9/28/2005	8.84	-	−11	−23	-	NT	NT	-
Thornton Cr, WA	4/11/1996	9/20/2005	9.44	-	-	-	-	-	NT	-
**Small streams, agricultural**										
Orestimba Cr, CA	8/5/1992	9/7/2005	13.09	−6	−9	−10	NT	5	NT	NT
Zollner Cr, OR	8/3/1993	8/3/2005	12.00	−16	9	−24	NT	−10	NT	NT
Rock Cr, ID	4/20/1993	9/6/2005	12.38	−5	-	-	NT	-	-	-
**Large streams, mixed land use**										
Merced R, CA	6/1/1993	6/22/2005	12.06	-	−8	−12	−27	-	−14	-
San Joaquin R, CA	6/1/1993	6/22/2005	12.06	NT	NT	−11	−11	NT	−11	−4
Willamette R, OR	9/1/1993	6/7/2005	11.76	−7	NT	−10	NT	−4	NT	-
Palouse R, WA	6/3/1993	8/2/2004	11.16	−16	-	-	-	-	−7	-
Snake R, ID	5/24/1994	7/19/2005	11.15	NT	-	-	−11	-	-	-

Notes: R, River; Cr, Creek. Dates are mm/dd/yy notation.

Significant downward trends in chlorpyrifos (−11%/year) and diazinon (−23%/year) were identified at Arcade Creek. The magnitude of the diazinon trend was smaller than the diazinon trend for the period 2000-2005 at the same site. The trend in chlorpyrifos for 2000-2005 was not statistically significant.

### Trends, Agricultural Sites, 1993-2005

Long-term trends were calculated at three agricultural sites: Orestimba Creek, California, Zollner Creek, Oregon, and Rock Creek, Idaho (Table 6). All pesticides had sufficient data to construct models at two or more sites. No significant trends in EPTC, simazine, or trifluralin were identified.

Long-term trends of atrazine at the same three sites were downward. The trend magnitude was small at Orestimba Creek (−6%/year) and Rock Creek (−5%/year). No trend was detected at these two sites during the short trend period. The long-term trend at Zollner Creek was −16%/year which was similar to the short-term trend (−12%/year). At all three sites, a larger downward trend was identified for the long trend period compared with the short trend period, which suggests that most of the decrease in the instream atrazine concentrations occurred during the 1990s.

Long-term trends in diazinon were downward at the two sites for which models were developed. The trend at Orestimba Creek was more negative during the short trend period (−46%/year) compared with the long trend period (−10%/year). At Zollner Creek the opposite was observed: no significant short-term trend was identified, but a large, significant downward trend (−24%/year) was identified during the long trend period. This suggests that a period of declining use in the 1990s was followed in the 2000s by a leveling off or an increase.

Significant long-term trends in both chlorpyrifos and metolachlor were identified at Orestimba Creek and Zollner Creek. Trends were in opposite directions at each site – chlorpyrifos was downward at Orestimba Creek (−9%/year) and upward at Zollner Creek (+9%/year) while metolachlor was upward at Orestimba Creek (+5%/year) and downward at Zollner Creek (−10%/year). Of these four cases, only the metolachlor trend at Orestimba Creek was significant for the short trend period (+20%/year).

### Trends, Large River Sites, 1993-2005

No upward long-term trends were detected among the five large river sites (Table 6). Fourteen of the 21 trend cases were downward; 7 were not significant. Consistent downward trends were detected for three pesticides: diazinon was down at three of the three sites with models, EPTC was down at three of the four sites with models, and simazine was down at three of the four sites with models. Atrazine was down at two of the four sites with models.

Long-term trends in diazinon were more consistent in magnitude and direction (−10 to −27%/year) compared with the short-term trends (−19%/year and two not significant). Trends in EPTC (−11 to −27%/year) and simazine (−7 to −14%/year) were identified at six of the eight large river sites compared with none at the small sites indicating important portions of the regional landscape are not represented by the small sites. No significant short-term trends in EPTC were identified compared with the long-term models, and while three of the four long-term simazine trends were downward, none of the short-term trends were downward and two showed increasing concentrations.

## Discussion of Trends

Positive trends indicating increasing concentrations of pesticides in a stream would be expected when significant changes in land use or cropping patterns have occurred, when novel pests arrive in a region, or when regulatory, marketing, or economic forces drive a widespread change in pesticide preference for a particular crop. Negative trends indicating declining concentrations of pesticides in a stream would be expected when a compound is phased out by regulatory action, where a watershed management plan such as a TMDL was instituted to reduce concentrations, when a product substitution has occurred, or with changes in water management that would limit transport, for example, conversion from flood or furrow irrigation to drip or sprinkler irrigation.

### Insecticide Trends: Chlorpyrifos and Diazinon

In 2000, the USEPA announced that it intended to phase out the OP insecticides chlorpyrifos and diazinon for all residential uses and for any other uses where children were likely to be exposed. In urban areas, the sale of chlorpyrifos-based products for most homeowner uses ceased in December 2001, postconstruction spot treatments for termite control were permitted until December 2002, and all preconstruction termite uses were canceled in December 2005 (USEPA, 2000). Indoor residential products containing diazinon were canceled in December 2002 and sales of outdoor residential products ceased in December 2004 (USEPA, 2006a). The December 2000 agreement between the USEPA and the registrant also specified reductions in diazinon production prior to the cancelation dates to speed removal of the product from the market.

Trends at the small urban sites reflect the phase-out of chlorpyrifos and diazinon. Between 2000 and 2005 trends in diazinon were downward at all sites where data were sufficient for modeling. No significant trends were identified for chlorpyrifos during this same time period. The lack of chlorpyrifos trends is attributed to the earlier date at which sales of chlorpyrifos-based residential lawn and garden products were canceled (December 2001) compared with those containing diazinon (December 2004). The step-down in diazinon production prior to December 2004 is probably responsible for the large trends observed during this time period. Had full-scale production been permitted up to the cancelation date, the trend would likely have been much smaller as only one growing season without product sales would have been modeled.

The phase out date of chlorpyrifos is likely reflected in the downward trend at Arcade Creek (−11%/year) for the long trend period (1996-2005). A downward trend in diazinon also was identified at the site for this trend period, but it was smaller (less negative) than the trend from 2000 to 2005. The difference in the diazinon trend magnitudes is consistent with use reductions occurring during the second half of the longer trend period and coincident with the regulatory phase out discussed earlier.

Agricultural reductions of chlorpyrifos and diazinon were also stipulated by the reregistration process and resulted in the cancelation of some uses and formulations and restrictions on the number of annual applications, application methods, and enhanced requirements for worker safety (USEPA, 2006a,c;). Agricultural uses of diazinon were restricted far more than chlorpyrifos uses, however, most of these restrictions did not take effect until after 2005.

Orestimba Creek, California, and San Joaquin River, California, were the only sites with significant non-urban downward trends in diazinon between 2000 and 2005. For the long trend period, downward trends in diazinon were identified at three non-urban sites in the San Joaquin River basin in California: Orestimba Creek, Merced River, and San Joaquin River. Downward trends in chlorpyrifos were identified at Orestimba Creek and Merced River; no significant trend was identified at San Joaquin River. All three sites have large amounts of agricultural land within their catchment areas (Table 1). Reductions in the use of chlorpyrifos, diazinon, and other OP insecticides in California starting in the mid-1990s are well documented (Epstein *et al.*, 2001; CalEPA, 2003, 2005; Oros and Werner, 2005; Zhang *et al.*, 2005). Reduction efforts were prompted by the recognition of lethal concentrations of these chemicals in the Sacramento and San Joaquin Rivers in the early 1990s (Kuivila and Foe, 1995; MacCoy *et al.*, 1995) followed by the listing of both rivers on the Clean Water Act Section 303(d) list of impaired streams in the mid-1990s. A TMDL plan to reduce diazinon and chlorpyrifos in the San Joaquin River was finalized in 2005. The trends at these sites are attributed to reductions in diazinon use in anticipation of the diazinon TMDL that was finalized for the California Central Valley in 2005 (CalEPA, 2005) rather than the phase out stipulated by the USEPA. Significant downward trends in diazinon would be expected at more agricultural sites if the modeling period extended to the present day.

The cause for the upward trend in chlorpyrifos at the Yakima River, Washington, during this time period could not be definitively ascribed to any known regulatory or land use changes during the modeling period, however two potential factors affecting chlorpyrifos use in the Yakima River basin were noted during the modeling period: (1) increasing chlorpyrifos use because of an increase in the planted acreage of silage corn in Yakima County (USDA, 2009b) or (2) increasing use in response to restrictions on other OP compounds such as azinphos-methyl (USEPA, 2001).

Long-term downward trends in diazinon were identified at two sites in the Willamette River basin in Oregon: Zollner Creek and Willamette River. These trends are likely not related to the diazinon phase-out stipulated by the reregistration eligibility decision (RED) as Zollner Creek is predominantly an agricultural catchment, and agriculture is the second largest land use in the Willamette River basin (after forested area) – most restrictions on agricultural uses of diazinon stipulated by the RED were phased in after 2005. Further, no significant trends in diazinon were identified at either site for the 2000-2005 trend period. Pesticide use data for Oregon are insufficient to understand these trends.

The widespread reductions of diazinon and chlorpyrifos in urban and agricultural settings have not come without increases in replacement products. Pyrethroid insecticides have become the most important replacement products in commercial, agricultural, and urban settings. Analyzing pesticide use data from CaDPR, Oros and Werner (2005) found that agricultural and nonagricultural uses of pyrethroid insecticides in the Sacramento and San Joaquin basins increased from around 23,000 kg in 1991 to more than 68,000 kg in 2003. In another analysis of CaDPR data, Epstein *et al.* (2001) focused on pyrethroid use in almond orchards in the San Joaquin River and Sacramento River basins during two time periods: 1992-1994 and 1995-1997. In all portions of their study area, they found large increases (41-1,445%) in the area treated with pyrethroid insecticides. Compared with OPs, pyrethroids are generally more toxic to fish and aquatic invertebrates and are more strongly sorbed to sediment (higher *K*_*d*_). As a result, sediment toxicity to benthic and infaunal organisms may be an important issue for consideration in future studies (Amweg *et al.*, 2005).

### Herbicide Trends: Atrazine, EPTC, Metolachlor, Simazine, Trifluralin

Upward trends in simazine were identified at three of the four small urban sites for the short trend period, 2000-2005. In contrast, only three trends (all upward) were identified among the other nine sites (small agricultural and mixed land use). Thornton Creek, Washington, was the only urban site without an upward trend. Urban pesticide use data are not available for any site, so specific sources and causes for the trends could not be identified.

The upward trend at the large river site, Palouse River, Washington, also might be associated with increasing urban uses. Simazine is not commonly used for agricultural purposes in the Palouse River basin nor is it used for weed control along road rights-of-way or for forest management (Wagner and Roberts, 1998; e-mail from Ray Willard, Landscape Architect, Washington State Department of Transportation to Henry Johnson, Hydrologist, December 7, 2009, Subject: Simazine use for rights of ways?; e-mail from Dan Hall, Whitman County Public Works to Henry Johnson, Hydrologist, December 2, 2009, subject: Simazine herbicide). The most likely source for simazine in the Palouse River is urban use in and around the cities of Moscow, Idaho, and Pullman, Washington.

**FIGURE 3:**
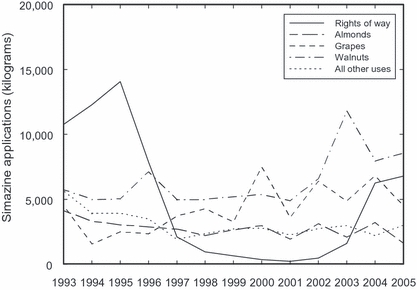
Reported Simazine Use in Counties Comprising the Sacramento River Basin.

Large increases in simazine applications to rights-of-way and walnut orchards were reported for counties in the Sacramento River basin between 2000 and 2005 (CaDPR, 2009) (Figure 3) and are likely a major cause of the observed trend at Sacramento River, Washington. The upward simazine trend at Granger Drain, Washington, is likely because of changes in agricultural use although the specific changes cannot be identified because pesticide use data is not available for the site. However, agriculture is the dominant use of the land in this catchment and the land classified as urban in this catchment (Table 1) is primarily roads, farms, and farming infrastructure. The only significant area of commercial and residential development in the catchment is approximately 15% of the area of the city of Granger (population 2,530).

No other consistent trends were identified among or across site groups for the other four herbicides for the 2000-2005 trend period. The opportunity to discuss forces behind changing stream concentrations is limited by a lack of pesticide use data across the study area.

Trends in atrazine were downward at all three small agricultural sites for the period 1993-2005 and at two of four large river sites for the period 1993-2005. No atrazine models were developed at the urban sites for the long trend period. Atrazine is often associated with corn because of the vast quantities applied to corn in the United States Midwest; however, in the four states in this study its use is more diverse and includes turf and forage grasses, forestry, Christmas tree plantations, and sorghum in addition to corn. The downward trends observed at the two large river sites (Willamette River, Oregon, and Palouse River, Washington) and at two of the small agricultural sites (Orestimba Creek, California, and Rock Creek, Idaho) were not observed during the shorter trend period (2000-2005) suggesting that the decrease in atrazine primarily occurred during the 1990s at these sites. At Zollner Creek, Oregon, however, the atrazine trend for the longer and shorter trend periods are both downward and of the same order of magnitude suggesting a single, continuous driver for the decreasing concentrations. A comparison of crops grown in the catchment and the crops for which atrazine is registered in Oregon, indicates that the most likely use of atrazine within the Zollner Creek basin is on Christmas tree plantations. The decreasing trend may reflect decreasing use or improved management.

**FIGURE 4:**
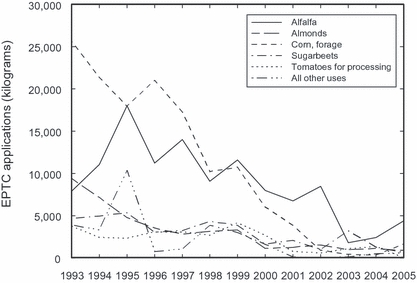
Reported EPTC Use in Counties Comprising the San Joaquin River Basin.

EPTC trends were downward at three of the four large river sites for which models were developed. Like atrazine, EPTC is registered for a relatively small number of crops in California, Oregon, Washington, and Idaho. Alfalfa, potatoes, corn, sugar beets, and beans are the dominant crops on which it is applied. Downward trends at the two large river sites in California reflect the decreasing use of EPTC reported to the California Department of Pesticide Regulation (CaDPR, 2009) (Figure 4). Large decreases in EPTC use on alfalfa and forage corn account for most of the change between 1992 and 2005. Presumably, farmers were replacing EPTC with one or more alternative products. The cause for the downward trend at Snake River could not be identified.

Trends in simazine at the large, mixed land use sites were downward at three of the four sites for the period 1993-2005; one site had no significant trend (Table 6). In contrast, the two significant trends at large river sites for the period 2000-2005 were upward. As with EPTC, two of the three downward trends are at sites in the San Joaquin River basin, and pesticide use data from California (CaDPR, 2009) are useful in discerning the causes. Simazine use in the five counties comprising the San Joaquin River basin decreased from 317,495 kg (mean use: 1993-1995) to 261,636 kg (mean use: 2003-2005). Use in the two counties comprising the Merced River basin was more modest: 43,881 kg (mean use: 1993-1995) to 40,372 kg (mean use: 2003-2005). Trends in both catchments were of a similar magnitude despite the disparity in the decrease between the two catchments. Because simazine is typically applied during the wet, winter months overland runoff is an important transport process. Applications occurring in areas adjacent to large impervious surfaces, such as along roadways, are more susceptible to runoff compared with applications occurring on farmland. In addition, road-side drainage ditches are designed to convey runoff to nearby waterways and may serve as conduits for chemicals applied along roadsides. For this reason and the fact that simazine use on farmland in the Merced River basin actually increased between 1993 and 2005, we suspect that much of the decreasing trend in simazine at the Merced River and San Joaquin River are because of reductions in rights-of-way applications.

The third downward trend in simazine was at the Palouse River. As was discussed for the upward trend identified for the 2000-2005 period of record at this site, changing urban use is the most likely cause for this trend. For the longer trend period no significant trends in simazine were identified at the two small agricultural sites (1993-2005) or at the two small urban sites (1996-2005). No further discussion is warranted because of a lack of pesticide use data at these sites.

Trends in pesticides result from regulatory changes, market forces driving consumers to choose a new or different product, changing land use (e.g., urbanization or introduction of new crops), improvements or restrictions to application methods, and improvements to water management (e.g., irrigation improvements or use of retention ponds and wetlands). Sometimes the cause for a trend is relatively clear, the phase out of OP insecticides in urban areas, for instance. Often however, spatial and temporal information about the driving forces are lacking at the catchment scale and interpreting the trends is not possible. It would be wrong, however, to confuse the inability to quantify catchment scale changes with the real effect such changes have on pesticides. Throughout the four-state region, pesticide use has been reduced on farms implementing alternative pest management strategies or participating in integrated pest management programs (Clark *et al.*, 1998; Brunner *et al.*, 2003; Zhang *et al.*, 2005). A correlation between pesticide use within a catchment and the amount exported has been noted in many studies (Capel *et al.*, 2001 and references therein for a thorough review). Additionally, local programs and demonstration projects throughout the region have encouraged farmers to replace their flood or furrow irrigation systems with sprinkler or drip systems to conserve water, soil, and minimize offsite chemical movement (North Yakima Conservation District, 1999; Sutton *et al.*, 2006; Orang *et al.*, 2008). An increase in the use of sprinkler or drip irrigation systems is associated with a reduction in the off-site transport of moderately to highly hydrophobic pesticides (Johnson, 2007), and retention of pesticides on the field or in the shallow soil promotes uptake, degradation, and infiltration (Klöppel *et al.*, 1997; Staddon *et al.*, 2001). Infiltration does not necessarily reduce pesticide transport to a stream (though it will delay delivery), and is associated with contamination of the underlying groundwater with pesticides and their metabolites (Böhlke, 2002; Gilliom *et al.*, 2006; Steele *et al.*, 2008). In 2004, the U.S. District Court restricted the application of 38 different pesticides near streams used by threatened or endangered salmon in California, Oregon, and Washington (USEPA, 2004a). A riparian buffer 18 meters wide was imposed for ground-based applications and 91 meters wide for aerial applications. No-spray buffers can reduce pesticide concentrations in streams (de Snoo and de Wit, 1998) by allowing for the deposition of spray drift (Schulz *et al.*, 2001; Woods *et al.*, 2001) and by infiltration, settling, sorption, and/or degradation (biotic and abiotic) of pesticides carried in overland runoff (Klöppel *et al.*, 1997; Staddon *et al.*, 2001). Lastly, changes in the production and use of pesticides and changes in land use have occurred since the early 1990s. In addition to changes in OP and pyrethroid insecticides already discussed, the production and use of EPTC, metolachlor, and trifluralin have seen decreases since 1993 (USEPA, 1997, 2004b). The direct impact of these decreases on the observed trends is confounded, however, by changing land use throughout the study area. For example, the acreage of corn planted for grain and sileage in Idaho and California have increased apace with the expanding dairy industry in those states (USDA, 2009a) potentially off-setting any decreasing trends because of reduced use of EPTC and metolachlor on those crops. Between 1990 and 2000, the population in Oregon, Washington, and Idaho grew by more than 20% and by 14% in California (United States Census Bureau, 2001). Assuming pesticide use by the new residents is similar to that of the 1990 population, urban pesticide use would have increased between 1990 and 2000.

## Applications of Modelfor Pesticide Management

The model used to estimate trends produced a variety of additional data, some of which can provide insights into spatial and temporal aspects of pesticide distribution, fate, and transport. The following sections provide a brief overview of some of those applications.

### Analysis of Peak Locations

**FIGURE 5:**
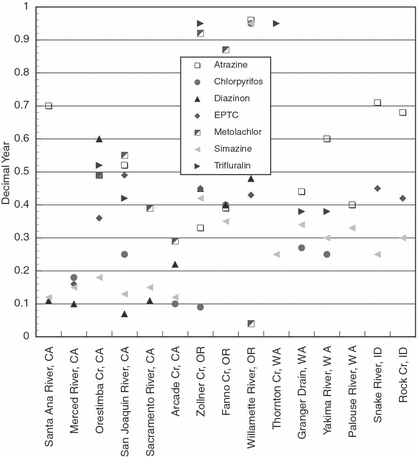
Annual Maximum of the Seasonality Function (*W*) for Each Site and Pesticide.

The peak of the seasonality function (*W*) identifies the time of year when maximum concentrations of pesticides are most likely to occur. The location of the peak concentrations for all pesticides is shown graphically in Figure 5. Although peak concentrations can occur at any time of the year in the western streams, some regional patterns are evident. Peak concentrations of diazinon, simazine, and chlorpyrifos at most streams in California occur early in the year, between about 0.05 and 0.25 decimal years (January 19-April 1). The peaks reflect the dormant season use of these pesticides on agricultural land (primarily orchards) and also winter applications of simazine to rights-of-way. The transport mechanisms and importance of pesticides applied during the winter in California has been documented in many studies (Kuivila and Foe, 1995; Domagalski *et al.*, 1997; Kratzer *et al.*, 2002; Dileanis *et al.*, 2003). The chlorpyrifos and diazinon peaks at Orestimba Creek, California, occur at 0.49 and 0.60 (around July 1 and August 1), respectively. Land use in the Orestimba Creek catchment is dominated by field and row crops rather than orchards, and the later peaks reflect the use of these chemicals to control pests during the growing season. A second large group of peaks occurs between about 0.30 and 0.55 decimal years (April 19-July 19), and is composed primarily of spring-applied herbicides. Two smaller groups of peaks can also be identified. One is a band of chlorpyrifos peaks narrowly clustered around 0.25 decimal years (April 1). The peaks coincide with the beginning of the irrigation season at the three sites (San Joaquin River, Granger Drain, and Yakima River) and may reflect the initial flushing of residual dormant sprays and preemergent applications by the first irrigation events of the year. The second small group begins around 0.87 decimal years (November 10) and continues into the new year until about 0.05 (January 19). It is confined to three sites in the Willamette Valley of Oregon and consists primarily of herbicides. The cluster of peaks coincides with the period of cool, wet weather that characterizes the late fall and winter in Western Oregon. Runoff from fall and winter storms transports residual summer pesticides and pesticides applied late in the year to control weeds in dormant vineyards, berry fields, and grass fields.

**FIGURE 6:**
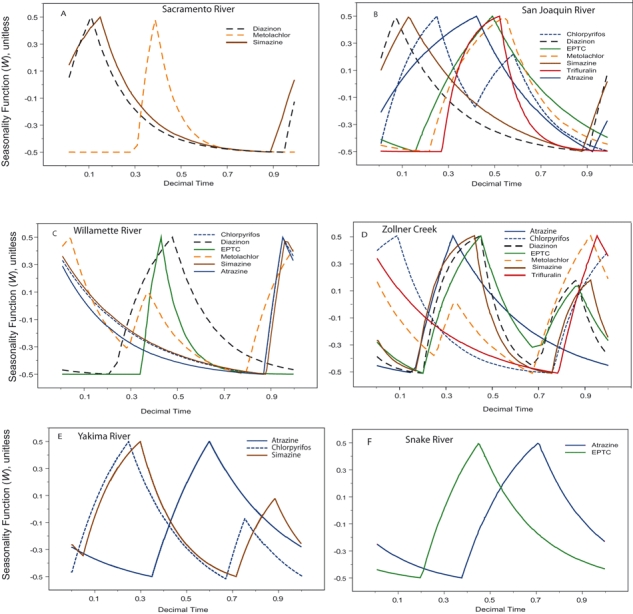
Seasonality Function (*W*) of All Pesticides at: (a) Sacramento River, (b) San Joaquin River, (c) Willamette, (d) Zollner Creek, (e) Yakima River, and (f) Snake River.

For a pesticide, the duration and maximum of the seasonal pulse varied among sites (Figure 6). A comparison of the distribution of all modeled pulses at a site can help assess the potential for instream exposure to elevated concentrations of pesticides. At some sites, such as Sacramento River, pesticide pulses are of short duration and confined to small portions of the year. Here, peaks of diazinon and simazine occur during the dormant spray season and a single peak for metolachlor occurs in the first third of the growing season. No peaks occur after the middle of the year, however, and the seasonality functions are approaching their annual minima (Figure 6). This indicates that the concentrations of the three modeled pesticides are approaching their seasonal minima despite the large amount of on-going agricultural activity. Highly controlled water use in rice fields (the basin's most extensive crop), widespread use of sprinklers and drip irrigation on nonrice agricultural land, and a lack of storm water runoff contribute to the generally good water quality throughout the remainder of the year.

In contrast to a site like the Sacramento River, some sites experience more frequent pesticide pulses, some having a longer duration, such as the San Joaquin River. Like the Sacramento River, dormant season peaks of diazinon, chlorpyrifos, and simazine occur at the San Joaquin River, but the pulses do not dissipate quite rapidly and a rapid succession of broad pulses follow and continue through the year (Figure 6). It is apparent from Figure 6 that the duration of the annual minimum is much shorter in the San Joaquin River compared with the Sacramento River.

The seasonal patterns at Zollner Creek in Oregon illustrate a third unique distribution of pesticide peak occurrence (Figure 6). Many pesticides at this site have two periods of peak occurrence – one in the spring and a second in the fall. The spring peak is similar to that observed at other sites and results from runoff of dormant sprays and preemergent herbicides. Pesticide runoff decreases in the summer because little rain falls, most of the region's agriculture is not irrigated, and the use of sprinklers and drip irrigation are common among those crops that do require supplemental water. The fall peak coincides with the end of the annual summer drought. Renewed rainfall transports recently applied and residual pesticides that were applied during the summer. Double peaks also are observed for four of the five pesticides at Fanno Creek, Oregon (not plotted), but for only one of the six pesticides (metolachlor) at Willamette River, Oregon (Figure 6), which receives water from both Zollner and Fanno Creek. The second peak for most pesticides likely is not expressed at the Willamette River site because most of the flow in the river originates in the Western and High Cascades where pesticide use is low, and this water dilutes out the pesticide's second peak.

In contrast to both the Willamette and California Central Valley rivers, rainfall runoff is not of great importance for the transport of pesticides at the Yakima and Snake Rivers. Pulses of pesticides are largely confined to the growing season (April-September, 0.25-0.75 decimal years) and are related to runoff of irrigation water and increased flow in tile drains. High flows in these rivers serve as diluting events and seasonal minima occur during the winter and during spring snowmelt. At the Yakima River site, simazine and chlorpyrifos peak in April shortly after irrigation water is first available to the growers, but drop quickly as irrigation water deliveries and runoff from snowmelt increase the flow (and therefore the dilution capacity) in the Yakima River. Atrazine peaks after snowmelt runoff has subsided. At the Snake River, EPTC peaks immediately after snowmelt runoff subsides, followed later in the summer by the peak in atrazine. Seasonal peaks of both pesticides are declining toward their annual minimum by the end of the irrigation season in mid-October (0.80 decimal years).

### Implications of Pesticide Peaks and Pulse Duration

Peak concentrations of pesticides analyzed for this study in the major rivers of this study coincide with periods of river use by threatened or endangered adult salmon and steelhead migrating up-river to spawn (Table 7). Pesticide exposure is not necessarily limited to the month of the annual peak. Elevated concentrations may be present for months before and after the annual peak, as indicated by the broad shape of many forms of the seasonality function shown in Figure 6. Exposure to pesticides by threatened or endangered salmonids in large rivers is not limited to adults returning to spawn. Large rivers also are used by maturing salmon and steelhead prior to their outbound migration to the ocean (National Marine Fisheries Service, 2008; Macneale *et al.*, 2010), although the period of use by temporarily resident fish is more difficult to ascertain than it is for returning adults. In one recent study, Teel *et al.* (2009) found subyearling juvenile Chinook salmon in the lower Willamette River and nearshore wetlands during the winter and spring, and coinciding with the annual peak of four of the six pesticides in this study reach.

**TABLE 7:** Seasonal Occurrence of Threatened or Endangered Adult Salmon and Steelhead Returning From the Ocean to Major Rivers in This Study. Shaded months indicate that returning adult fish may be present. Letters indicate the month in which the peak pesticide concentration occurs

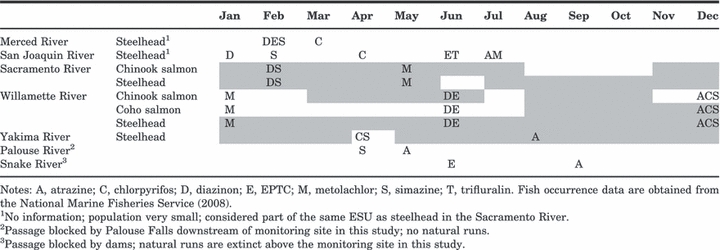

### Time Series Estimates of Concentration

The primary function of the model (LOADEST) that was used to estimate trends is to estimate contaminant loads (mass/time) and concentrations (mass/volume) at daily, monthly, and annual time steps. Simulated daily pesticide concentrations are potentially a useful tool in evaluating compliance with water quality criteria while estimates of loads are useful in evaluating changes in pesticide management and use within a catchment.

**FIGURE 7:**
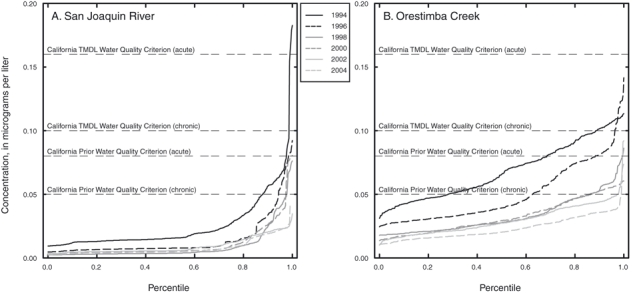
Percentile Plot of Simulated Diazinon Concentrations and TMDL Targets for the Nested Sites (a) San Joaquin and (b) Orestimba. For legibility, simulated concentrations are only presented for even-numbered years; the concentration percentiles in odd-numbered years are similar to those in the preceding and subsequent years.

In 2002, the lower San Joaquin River was listed on the federal 303(d) list as impaired for beneficial uses because of toxicity due to OP insecticides and as a result, a TMDL was implemented (CalEPA, 2005). Simulated concentrations of diazinon from the model are shown in Figure 7 for the nested basins, Orestimba Creek and San Joaquin River, along with two different water quality objectives. Because of the fact that the simulated concentrations are daily mean values, the frequency of exceeding the acute toxicity criteria (generally set as an hourly or daily maximum) may be underestimated, while the frequency of exceeding the chronic toxicity criteria (generally set as a multiday maximum) may be overestimated. Diazinon concentrations were above the initial chronic criteria at Orestimba Creek for more than half the year in 1993, 1994, and 1995. At the San Joaquin River, concentrations exceeded the initial chronic criteria for 10% of the year in 1993 and 1994. In 2005, the water quality objectives were recalculated given new information on toxicity (CalEPA, 2005). From 1998 to the end of this period of record, diazinon concentrations are below the TMDL targets 100% of the time, according to the model results.

## Conclusions

Significant changes in concentrations of many pesticides in streams of California and the Pacific Northwest were identified during the course of this study. Pesticide trends were calculated for two time periods at most sites: a common period spanning 2000-2005 was used at all 15 sites, and a longer period beginning in 1993 or 1996 and extending to 2005 was used at 10 sites. Trends at a site often were not consistent between the two time periods, reflecting the dynamic nature of pesticide use and management in the region. However, cohesive regional patterns for some pesticides were identified. Downward trends in diazinon were identified at five of the eight sites between 2000 and 2005 and at six of the six sites between the mid-1990s and 2005. These decreasing trends were related to regulatory and management actions enacted by state and federal agencies. Between 2000 and 2005, simazine concentrations increased or did not significantly change. In contrast, simazine trends either were downward or not significant during the longer trend period. In fact, during the longer trend periods, most trends either were not significant or were downward; only two pesticide trends (out of 42) were upward. Downward trends in EPTC, metolachlor, and trifluralin for the long trend period are consistent with national production and use reported by the USEPA. Despite this correlation, the driving forces behind the trends (or lack thereof) generally were not identified because of incomplete information on changes in chemical use, land use, and chemical management at the catchment scale.

Concerns with pesticides in the streams studied in this paper often are related to pesticide exposure by developing juvenile and migrating adult fish, particularly threatened and endangered salmonids. Output from the log-linear multivariate regression model used to estimate trends provides insight into the time of year when peak concentrations occur, the duration of elevated concentrations, and the hydrological conditions when they occur. The model can also provide estimates of concentrations and statistical confidence on those estimates that may be useful to resource managers in catchments with TMDLs or other regulatory mandates to reduce pesticides in streams.
